# RNA-seq analysis of *Drosophila* clock and non-clock neurons reveals neuron-specific cycling and novel candidate neuropeptides

**DOI:** 10.1371/journal.pgen.1006613

**Published:** 2017-02-09

**Authors:** Katharine C. Abruzzi, Abigail Zadina, Weifei Luo, Evelyn Wiyanto, Reazur Rahman, Fang Guo, Orie Shafer, Michael Rosbash

**Affiliations:** Howard Hughes Medical Institute and National Center for Behavioral Genomics,Department of Biology, Brandeis University, Waltham, United States of America; Charité—Universitätsmedizin Berlin, GERMANY

## Abstract

Locomotor activity rhythms are controlled by a network of ~150 circadian neurons within the adult *Drosophila* brain. They are subdivided based on their anatomical locations and properties. We profiled transcripts “around the clock” from three key groups of circadian neurons with different functions. We also profiled a non-circadian outgroup, dopaminergic (TH) neurons. They have cycling transcripts but fewer than clock neurons as well as low expression and poor cycling of clock gene transcripts. This suggests that TH neurons do not have a canonical circadian clock and that their gene expression cycling is driven by brain systemic cues. The three circadian groups are surprisingly diverse in their cycling transcripts and overall gene expression patterns, which include known and putative novel neuropeptides. Even the overall phase distributions of cycling transcripts are distinct, indicating that different regulatory principles govern transcript oscillations. This surprising cell-type diversity parallels the functional heterogeneity of the different neurons.

## Author summary

Organisms ranging from bacteria to humans contain circadian clocks. They keep internal time and also integrate environmental cues such as light to provide external time information for entrainment. In the fruit fly *Drosophila melanogaster*, ~150 brain neurons contain the circadian machinery and are critical for controlling behavior. Several subgroups of these clock neurons have been identified by their anatomical locations and specific functions. Our work aims to profile these neurons and to characterize their molecular contents: what to they contain and how do they differ? To this end, we have purified 3 important subgroups of clock neurons and identified their expressed genes at different times of day. Some are expressed at all times, whereas others are “cycling,” i.e., expressed more strongly at a particular time of day like the morning. Interestingly, each circadian subgroup is quite different. The data provide hints about what functions each group of neurons carries out and how they may work together to keep time. In addition, even a non-circadian group of neurons has cycling genes and has implications for the extent to which all cells have or do not have a functional circadian clock.

## Introduction

Nearly all organisms possess a circadian clock, which allows for the adaptation and anticipation of the daily oscillations of day (light) and night (dark). The circadian clock of *Drosophila melanogaster* drives a 24-hour locomotor activity rhythm, which includes bouts of morning and evening activity. This rhythmic behavior is controlled by a molecular clock, which includes transcriptional negative feedback loops that are conserved from insects to mammals. Clock (CLK) and Cycle (CYC) form a heterodimeric transcription factor that functions as the central circadian transcriptional activator. CLK/CYC activates the expression of two transcription factor genes, *timeless* (*tim*) and *period* (*per*) in the late morning. TIM and PER enter the nucleus in the early night, inhibit CLK/CYC driven transcription, and sequester CLK/CYC until morning. Once released, CLK/CYC start the cycle over again by activating *tim* and *per*. This negative feedback leads to oscillating gene expression for *per* and *tim* as well as many other CLK/CYC controlled genes. Two other CLK/CYC transcriptional target genes, *Vrille* (*vri*) and *par domain protein 1* (*pdp1*) encode transcription factors that form a second circadian feedback loop. The cyclical expression of many different genes provides temporal control of different behaviors or outputs of the clock; they include for example feeding and sleep (reviewed in [1], [2]).

The molecular clock is expressed in ~150 clock neurons in the *Drosophila* brain, which function together to regulate many of these circadian behaviors. These neurons are classified based upon their anatomical location (reviewed in [3, 4]). There are dorsal neurons that are divided into three groups: DN1s, DN2s and DN3s. There are also lateral neurons (LNs), which can be subdivided into 4 groups. They include the lateral posterior neurons (LPN; 3 neurons), dorsal lateral neurons (LNds; 6 neurons), and two groups of ventral lateral neurons: the small ventral lateral neurons (s-LNvs; 5 neurons) and the large ventral lateral neurons (l-LNvs; 4 neurons). The LNs can also be subdivided based on expression of the neuropeptide, PDF (pigment dispersing factor). The PDF^+^ lateral neurons consist of all of the LNvs except the 5^th^ small LNv. PDF^-^ lateral neurons consist of all the LNds plus the 5^th^ small LNv. The PDF^+^ LNvs are considered to be the major fly pacemaker neurons as they are sufficient to drive rhythmic locomotor behavior in the absence of light cues [5, 6].

Like in flies, an anatomically restricted region of the mammalian brain serves as the circadian central pacemaker. This is the suprachiasmatic nucleus (SCN), a subregion of the hypothalamus that contains ~15,000 neurons (in mouse). The SCN has two main regions: the ventrolateral “core,” which expresses vasoactive intestinal polypeptide (VIP); and the dorsolateral “shell,” which expresses arginine vasopressin (AVP). Although the core and shell provide a simple anatomical framework, the SCN is complicated: different regions oscillate in different phases, express scores of different neuropeptides and project to unique target areas of the brain [7–9].

A key question in both systems is how brain circadian neurons work together to drive complex circadian behaviors. Due to the relative simplicity of the *Drosophila* system, much more is known about the fly circadian network. The PDF neurons, the l-LNvs and s-LNvs, are probably part of the primary light-input pathway to the clock. They receive light information directly via the intracellular presence of the blue-light photoreceptor Cryptochrome (CRY) as well as indirectly via photoreceptors of both the compound eyes and the H-B eyelets [10–13]. PDF release by the LNvs is critical for communicating time of day signals to the LNds and DN1s as well as to the non-circadian LK/LK-R neurons [14–16].

A subset of the LNds, the 3 Cry+ LNds well as the 5^th^ small PDF^-^ LNv, are important for controlling evening anticipatory behavior and are therefore referred to as evening cells [17–19]. However, their role is not limited to driving evening activity as they can also modulate morning activity [20]. This is because silencing them leads to a strong decrease in both morning and evening locomotor activity, and other experiments from our lab indicate that the LNds are general activity-promoting neurons [19].

The DN1s are intriguing. A recent study illustrates that the circadian clock controls daily changes in DN1 membrane excitability [21]. This cell-autonomous control is then modulated by effects from the circadian network. For example, PDF signaling from the LNvs to the DN1s is important for arousal in the morning [22–24]. The DN1s then release the neuropeptide, Dh31, to promote awakening at dawn [25]. Later in the day however, DN1s send inhibitory signals to the LNds and LNvs to promote the siesta and nighttime sleep [26–28]. Not surprisingly, the DN1s contact several groups of neurons to carry out these multiple functions: the pars intercerebralis (PI), the LNds and the LNvs [19, 23, 24, 29].

To learn more about these three important groups of circadian neurons and what molecules may be important for their functions, we used RNA-sequencing (RNA-seq) to profile the transcriptomes of isolated PDF^+^ lateral neurons (referred to subsequently as LNvs), PDF^-^ lateral neurons (LNds plus including 5^th^ small PDF^-^ LNv; referred to subsequently as LNds) and DN1s. We also assayed dopaminergic neurons (referred to as TH; tyrosine hydroxylase) as a non-circadian outgroup. This profiling was done “around the clock” to address the temporal (circadian) regulation of gene expression. First, we identified both common as well as group-specific transcripts and then identified among them known and putative neuropeptides. They could facilitate intra-circadian network communication and/or communicate with neurons outside of the circadian network to drive output behaviors. Second, we identified cycling transcripts in each neuronal group. The low level of core clock gene expression in dopaminergic neurons indicates that cell-autonomous clock function may not be ubiquitous in the fly brain. Nonetheless, a small number of cycling transcripts are identified in TH neurons. In the four different circadian neuronal groups, cycling gene expression was almost completely distinct, which resembles what has been reported for mammalian cells and tissues. In addition the phase distribution of these cycling clock neuron transcripts was strikingly different in the LNvs, suggesting that distinct mechanisms determine the phase of transcript cycling within different clock neurons.

## Results

### Profiling the transcriptome of specific neuronal populations

To compare specific *Drosophila* circadian neuron subsets, we sequenced the transcriptomes of 3 well-described groups of circadian neurons: the LNds (LNds plus 5^th^ PDF- LNv), LNvs (small and large PDF^+^ cells) and a subset of the DN1s. We also sequenced a non-circadian outgroup, dopaminergic cells (TH cells; ~120 neurons per brain). Neuron groups were labeled with GFP using specific *GAL4* drivers and manually isolated with 3 rounds of fluorescent cell sorting (see Methods and [30]). Every sample contained 50–100 cells, which yielded approximately 200-500pg of total RNA; this was amplified to make RNA libraries for deep sequencing (see Methods and [30]). Two independent sets of circadian time courses were performed for each group at 4 hour intervals to identify cycling transcripts (see below). Experiments were performed in light:dark (LD) conditions to maximize comparisons between cycling gene expression and circadian behavior, which is more robust in LD than in DD. We also combined data from both replicates of 6 circadian time points, pooling all 12 samples from each neuron group, to address cell type-specific gene expression without regard to circadian time.

**Fig 1:**
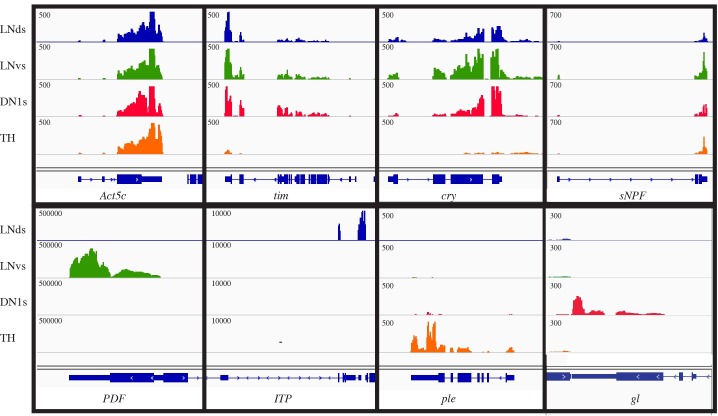
Sequencing libraries reflect the transcriptomes of LNds, LNvs, DN1s and TH neurons. Sequencing results from LNds (blue), LNvs (green), DN1s (red), and TH cells (orange) are shown for a variety of genes. The housekeeping transcript, Act5c, shows similar levels in all four neuronal groups. The two circadian transcripts, tim and cry are found in the three groups of circadian neurons but are expressed at very low levels or not at all in TH cells. The transcript of the neuropeptide sNPF is found in all four subgroups of neurons. The transcripts encoding neuropeptides PDF and ITP are found in the LNvs and LNds, respectively. The tyrosine hydrolyase transcript, *ple*, is strongly expressed in TH cells and the transcription factor, *gl* (glass) is expressed specifically in the DN1s. Note that there is some 3’-bias in these libraries; there is generally more signal on the 3’-end of the gene than the 5’-end. Y-axis scale is in reads/million total reads.

As expected, the circadian genes *timeless* (*tim*) and *cryptochrome* (*cry*) are strongly expressed in all three groups of circadian neurons but poorly expressed in non-circadian dopaminergic neurons (Fig 1). Also as expected, a control gene, *actin* (*Act5c*), is expressed approximately equally in all 4 groups of neurons. Previous studies have shown that the neuropeptides *PDF* and *ITP*, the dopamine biosynthesis gene *ple*, and transcription factor *gl* are expressed in LNvs, LNds, TH cells and DN1s, respectively [31–34]. Our deep sequencing results confirm these observations: *PDF* is expressed solely in the LNvs, *Itp* mRNA is highly expressed only in the LNds, *ple* transcripts are enriched in TH cells and *gl* mRNA is found exclusively in the DN1s (Fig 1). As evidenced by the presence of the s-LNv-specific transcript sNPF (Fig 1; [32], the LNv samples contains the large cells (l-LNvs) as well as the harder to isolate small cell population (s-LNvs).

Some transcripts on the other hand show unexpected profiles. For example, the neuropeptide sNPF is expressed not only in the small LNvs but also the LNds and TH cells, but it has not been detected in DN1s [35, 36]. However, sequencing data indicate that *sNPF* transcripts are present in DN1s as well as the 3 expected locations (Fig 1). There are a few other discrepancies between transcript detection in sorted cells and previous immunostaining results (see Discussion). Nonetheless, the good correlation with previously defined neuron-specific factors suggests that the RNA sequencing libraries reflect the transcriptomes of these four neuronal groups.

### Transcriptome differences suggest functional differences

We then used the transcriptional profiling results to address two questions. First, do the three groups of circadian neuron have shared transcripts beyond the core clock mRNAs? These additional transcripts may play some common role in the different clock neurons, for example circadian timekeeping like the core clock mRNAs. Second, are there transcripts enriched in a single circadian group, which could provide insight into the more specialized functions of that group [28]?

To address the first question, we identified transcripts that are more highly expressed in at least two of the three circadian groups relative to TH neurons (Table 1; see below for an explanation of why we did not require enrichment in all 3 groups). 18 transcripts are enriched by this criterion (greater than 5-fold enrichment and p-value <0.05 in Anova Tukey HSD post-hoc test with Benjamini Hochberg correction; see Methods).

**Table 1:** Common transcripts in circadian neurons.

	Fold Enrichment LNvs vs. TH (log2)	Fold Enrichment LNds vs. TH (log2)	Fold Enrichment DN1s vs. TH (log2)
*cry*	4.04	2.78	4.11
*vri*	3.75	2.68	2.93
*tim*	2.98	2.43	2.83
*npf*	4.54	4.46	6.51
*CG17777*	6.48	6.46	5.81
*CG13054*	6.35	6.36	6.80
*Tdc2*	4.10	3.23	3.56
*vib*	3.91	2.76	4.37
*Tbh*	4.94	2.33	2.09
*Hr51*	3.90	3.37	1.00
*CG11221*	2.40	2.54	1.02
*per*	2.34	LR	3.01
*Clk*	3.33	LR	3.80
*Pdfr*	3.32	LR	4.79
*Dh31*	4.08	0.28	4.04
*MCPH1*	2.84	0.78	2.43
*CG31714*	2.81	0.77	2.78
*CG6912*	LR	8.54	8.31

Transcripts enriched in two or more groups of the circadian neurons relative to the non-circadian control (TH) are listed. Fold-enrichment is shown in log base 2. To be enriched, the transcript must be greater than 5-fold higher in the circadian neurons than in the TH neurons. Those values shown in pink do not meet cutoffs for enrichment. The core clock genes are enriched in at least 2/3 classes of circadian neurons. Both *per* and *Clk* are expressed at low levels in LNds and do not meet expression thresholds for the comparison. LR = low reads.

As expected, almost all core clock genes are present among these 18 genes. c*ry*, *vri*, and *tim* are enriched in all three groups, but *per* and *Clk* are only enriched in two. This is because *per* and *Clk* mRNAs are not sufficiently expressed in LNds to reach the required threshold of 10 average reads/million (Table 1, LR low reads). Several other enriched genes have also been implicated in circadian processes, e.g., the neuropeptide Dh31 [25] as well as the transcription factor unfulfilled or HR51[37, 38], whereas others function in a variety of different processes. They include the neuropeptide (*npf*) as well as two genes involved in octopamine synthesis (*Tdc2* and *Tbh*). In addition, they could also contribute to aspects of circadian function carried out similarly by all three circadian groups.

**Fig 2:**
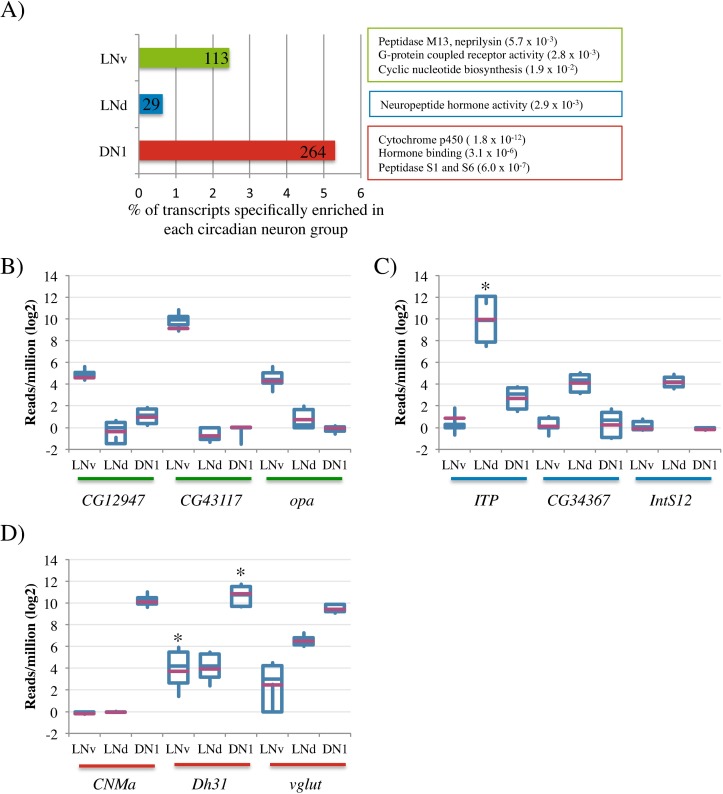
Transcripts enriched specifically in one group of circadian neurons. A) The percentage of transcripts specifically enriched in each of the circadian neuronal groups is represented in a bar graph. LNvs are shown in green. LNds are shown in blue. DN1s are shown in red. The absolute number of enriched genes is indicated in each bar. Results of gene ontology analysis (GO) are included. B, C, and D) Boxplots showing the expression levels of some of the most significantly enriched transcripts in the LNvs (B), LNds (C) and DN1s (D). The purple bar indicates the mean. The asterix denotes those transcripts that show high variability due to cycling transcripts levels.

To address neuron-specific functions, we identified transcripts that are more abundant in one group of circadian neurons relative to the other 2 groups (Fig 2A; greater than 2-fold enrichment, p-value <0.05 in Anova Tukey HSD post-hoc test with Benjamini Hochberg correction; see Methods).

About 2.5% of the LNv transcriptome, 113 genes, meet this criterion (S2 File). They include genes previously shown to be preferentially expressed in LNvs, e.g., the neuropeptide *PDF*, the transcription factor *dimmed*, the translation factor *twenty-four* (*tyf*) as well as the octopamine receptor, *oamb* [39–43]. In addition, three highly significant LNv-enriched transcripts include two of unknown function (CG12947 and CG43117) as well as a transcript encoding the putative Clk coactivator *opa* (Fig 2B; [44]). Gene ontology analysis (GO; see Methods) indicates that genes encoding G-protein coupled receptors (4 GPCR genes; *Dh31-R*, *MsR1*, *AstC-R2*, *CG13229*), genes involved in cyclic nucleotide biogenesis (3 genes), and genes encoding members of the Peptidase M13, neprilysin family (3 genes; *Nep1*, *Nep2* and *Nep3*) are overrepresented among the list of LNv mRNAs. These functions are consistent with our proposal that the LNvs integrate environmental information (GPCRs) and transmit that information to the rest of the circadian network [19], i.e., requiring signal transduction (cyclic nucleotide biogenesis) and neuropeptide processing (peptidases).

Although a much smaller fraction of the LNd transcriptome is enriched compared to the other two groups of circadian neurons (<1%, 29 transcripts; S2 File), several known LNd-specific transcripts were identified. They include the acetylcholine biosynthetic transcript *ChAT* and the neuropeptide *ITP*; their products have both been identified in LNds by immunohistochemistry [32]. In addition, the transcripts encoding the bHLH transcription factor *CG34367* and a component of the integrator complex that processes snRNAs, *IntS12*, are significantly LNd-enriched (Fig 2C; S2 File). GO analysis indicated that transcripts involved in neuropeptide hormone activity are enriched in LNds (3 genes; *hug*, *ITP*, *Dh44*). The expression of *Dh44* is surprising as this neuropeptide is reported to be absent from these neurons ([24]; see Discussion).

About 5% of the DN1 transcriptome, 264 transcripts, is enriched compared to LNvs and LNds (S2 File). The neuropeptide hormone Dh31 was recently reported to be strongly expressed in DN1s [25], and its transcript is indeed expressed much more highly in the DN1s than in the other two circadian neuron groups (~100-fold; Fig 2D). In addition, two transcripts known to be expressed in the DN1s, the transcription activator, *gl*, and the glutamate vesicular transporter, *Vglut*, are enriched ([34] [45]; Fig 1, Fig 2D; see Discussion). GO analysis indicates that genes in the cytochrome p450 family (15 genes), genes encoding proteins involved in hormone binding (7 genes), and genes encoding S1 and S6 peptidases (10 genes) are overrepresented in the more highly expressed DN1 genes (Fig 2A). Other highly enriched DN1 transcripts include the neuropeptide *CNMa* (Fig 2D).

**Fig 3:**
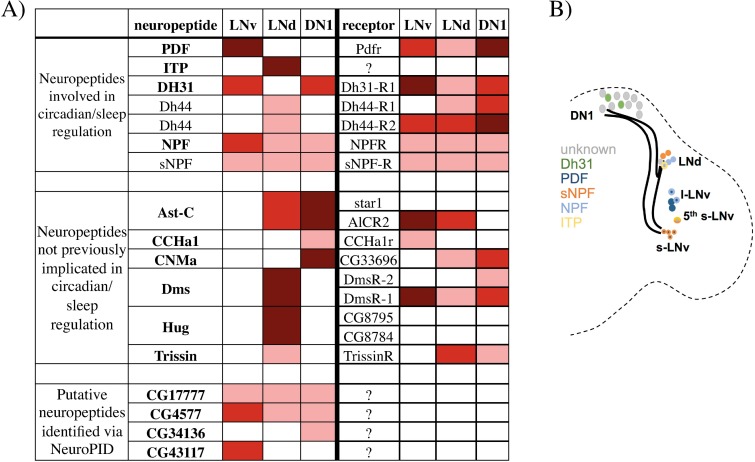
Known and putative neuropeptide and receptor encoding transcripts found in the three circadian neuronal groups. A) Table shows neuropeptides listed on the left side and their receptors (if known) on the right side. Those in bold were identified as pro-neuropeptides by NeuroPID (see Methods). Color indicates the level of expression in each group of neurons. For neuropeptides: dark red (>1000 reads/million), red (between 500 and 1000 reads/million) and pink (between 20 and 500 reads/million). Receptor transcripts are generally found at much lower levels: dark red (> 30 reads/million), red (between 10 to 30 reads/million) and pink (5 to 10 reads/million). Those shown in bold were identified via NeuroPID (see Methods). B) The cartoon shows the organization of the circadian neuronal network in *Drosophila* and the expression pattern of those neuropeptides known to have a role in the circadian system.

Because neuropeptides feature prominently in this analysis, we examined this class of genes in more detail. Transcripts encoding neuropeptides known to be expressed in the circadian network were identified (Fig 3A), and the localization of these neuropeptides within the circadian neurons is summarized in a cartoon (Fig 3B). In addition, transcripts for several neuropeptides not known to be expressed in circadian neurons were identified in LNds (hugin, Dms, Trissin, and Ast-C) and DN1s (Ast-C, CCHA1, and CNMa). Receptor mRNAs for some neuropeptides were also expressed in the circadian network (Fig 3A), suggesting that they may act in part within this network.

In addition to known neuropeptides, we also noticed a number of short, non-intron-containing transcripts that are enriched in circadian neurons; these features are common in neuropeptide genes. To further explore this possibility, NeuroPID was used to analyze the predicted proteins encoded by these mRNAs [46]. NeuroPID examines a peptide sequence for signal peptides and cleavage sites characteristic of pro-neuropeptides (see Methods). As proof of principle, NeuroPID successfully identified many known neuropeptide precursors among the large number of transcripts enriched in circadian neurons (Fig 3A; bold). Prominent exceptions are mRNAs for sNPF and Dh44, which were not identified. NeuroPID also identified putative novel pro-neuropeptides, some of which scored similarly to well-characterized neuropeptides (Fig 3A; first column). For example, CG17777 is a putative signal peptide-containing pro-neuropeptide identified by NeuroPID. It is expressed in all three circadian neuron groups and is also enriched (Table 1). Two of the most abundant transcripts enriched in LNvs, CG43117 and CG4577, also encode putative proneuropeptides by these criteria (Fig 2B and Fig 3A).

### Distinct groups of cycling genes identified in LNvs, LNds, DN1s and dopaminergic neurons

Transcriptome profiling of *Drosophila* heads has identified many cycling transcripts [47–50]. However, there may be additional genes under circadian control only within individual neuron groups. To address this possibility in a comprehensive manner, cycling transcripts were identified in the four groups of neurons. As mentioned above, 2 independent 6 time point circadian RNA samples were purified and sequenced from each group, and they were analyzed using both JTK cycle (p-value < 0.05) and fourier analysis (F24 score > 0.5; see Methods for additional criteria). Genes that encode cycling transcripts with both methods were defined as high confidence cyclers (HC cyclers), and genes that cycle with only one method were low confidence cyclers (LC cyclers).

The two methods identified between ~150–300 HC cyclers in each circadian group and many fewer in TH neurons, i.e., 249, 303, 185, and 31 HC cyclers in LNvs, LNds, DN1s, and TH neurons, respectively; S3 File). As ~30% of the cyclers identified by fourier analysis are also identified with JTK cycle (S1 Fig), the HC criterion has a much lower false positive rate. The stringent HC criterion may explain why we observe so few cyclers compared to mammalian studies, especially compared to a recent SCN study [51]. However, it is generally the case that flies have fewer cycling transcripts than mammals [52, 53]. Only 4% of these HC cyclers were previously identified as cycling head transcripts using similar methods and fly lines [50]. This comparison suggests that many cycling circadian neuron transcripts are indeed invisible in studies of more heterogeneous tissues like the fly head and fly brain because they are neuron-specific.

**Fig 4:**
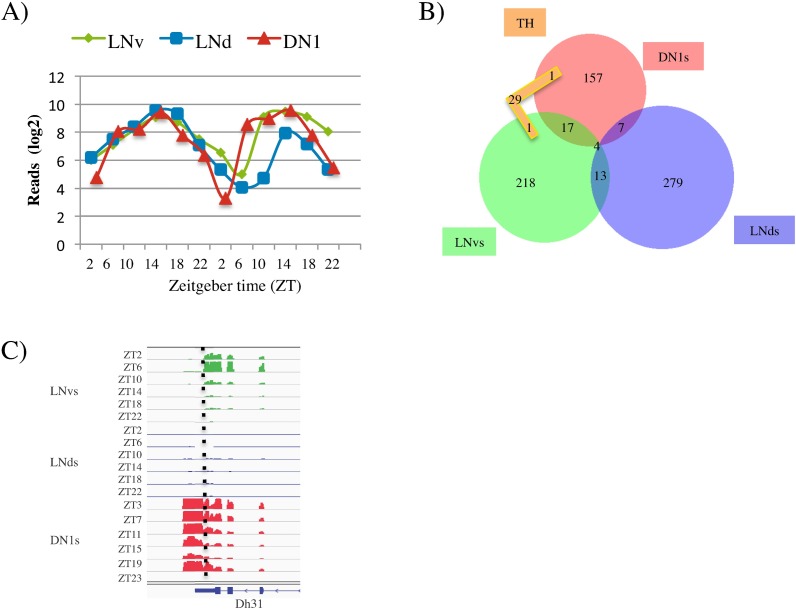
Most cycling gene expression is specific to one group of circadian neurons. In all figures, LNvs are shown in green, LNds are shown in blue, DN1s are shown in read, and TH are shown in orange. A) *Timeless* (*tim*) cycles in all three neuronal groups with similar phase and expression level. Transcript levels are represented as reads/million total reads in a log base 2 scale. Two independent six timepoint datasets are concatenated to show cycling. B) Overlap of the high-confidence (HC) cycling transcripts found in the four neuronal groups. Only 4 HC cycling transcripts are in common in the 3 groups of circadian neurons. C) Dh31 is a HC cycling transcript in both the LNvs (green) and DN1s (red) with peak expression in the morning. The dotted line denotes the 3’end of the LNv specific Dh31 isoform. The isoform of Dh31 expressed in DN1s has an extended 3’-UTR. See also Fig 3, S1 Fig and S2 Fig.

We first examined the known CLK/CYC core clock target genes: *tim*, *per*, *vri*, *and pdp1*. To identify all 4 transcripts as cyclers in the three groups of circadian neurons required the LC criterion. This emphasizes the stringent nature of the HC criterion, which is useful for numerical comparisons but not necessarily for identifying individual cycling transcripts because of false negatives (Fig 4A and Table 2). The TH neurons express low levels of these core clock genes, and only *tim* was identified there as a cycling (LC) transcript.

**Table 2:** Transcripts cycling in two or more groups of circadian neurons.

	Cycles LNv	Cycles LNd	Cycles DN1	Gene Ontology
*tim*	+	+	+	Circadian rhythm
*vri*	+	+	+	Circadian rhythm
*Pdp1*	+	+	+	Circadian rhythm
*per*	+	+	+	Circadian rhythm
*SK*	+	+	+	Small conductance potassium channel activity
*CG31324*	+	+	+	Unknown
*CG14086*	+	+	+	Unknown
*ptip*	+	+	+	Histone methylation
*NAAT1*	+	+	+	Neurotransmitter; sodium symporter activity
*ATPsynC*	+	+	+	ATP hydrolysis coupled proton transport
*CG31475*	+	+	+	Neuron projection morphogenesis
*CG13995*	+	+	+	G-protein coupled receptor
*CG13054*	+		+	Unknown
*CG17777*	+		+	Unknown
*CG18011*	+		+	Unknown
*CG32369*	+		+	Proteolysis
*CG6073*	+		+	Unknown
*USP8*	+		+	Protein deubiquitination
*MCT1*	+		+	Transmembrane transport
*CG31183*	+		+	cGMP-mediated signaling; cyclic nucleotide biosynthetic process
*Dh31*	+		+	Neuropeptide signaling pathway
*Orc2*	+		+	Neurogenesis; chromatin silencing
*twit*	+		+	Regulation of neurotransmitter secretion
*CG14400*	+		+	Unknown
*Dyrk2*	+		+	Protein phosphorylation; response to light stimulus
*Tim8*	+		+	Protein targetting to mitochondria
*cwo*		+	+	Circadian rhythm
*CG43902*		+	+	Unknown
*zye*		+	+	Actin filament organization
*cals*		+	+	Synaptic transmission
*mtm*		+	+	Endocytic recycling
*scra*		+	+	Neurogenesis
*CG32313*	+	+		Unknown
*CG32000*	+	+		Cation transport
*CG9005*	+	+		Response to endoplasmic reticulum stress
*CG11576*	+	+		Unknown
*CG1971*	+	+		Unknown
*CG7852*	+	+		Regulation of Rab protein signal transduction
*Tm1*	+	+		Dendrite morphogenesis
*CG43143*	+	+		Protein phosphorylation
*sli*	+	+		Neuron differentiation; axon midline choice point recognition
*CG11486*	+	+		Nuclear transcribed mRNA poly(A) tail shortening

List of genes whose transcripts cycle in at least two of three groups of circadian neurons. CLK/CYC controlled clock genes are listed at the top. If the transcript was a low-confidence cycler in the third group of circadian neurons, this is denoted with a small “+” sign. The molecular function proposed by gene ontology analysis is listed for each gene.

Even using LC as well as HC criteria, only 12 cycling mRNAs (the 4 core clock genes plus 8 others) are common among all 3 circadian neurons. These transcripts are involved in diverse processes, from histone methylation to neuron morphogenesis (Table 2). A total of 30 additional transcripts (~6% of the total) were identified as HC cyclers in two circadian groups (Table 2 and Fig 4B). The list includes several genes previously shown to impact circadian rhythms and/or sleep (e.g. *cwo*, *Usp8*, and *Dh31*). Although the levels of *Dh31* mRNAs are much higher in DN1s than in LNvs, these neuropeptide-encoding transcripts were identified as HC cyclers in both sets of neurons. Interestingly, *Dh31* expresses a different transcript isoform in LNvs, where it has a much shorter 3’-UTR (Fig 4C; see Discussion).

**Fig 5:**
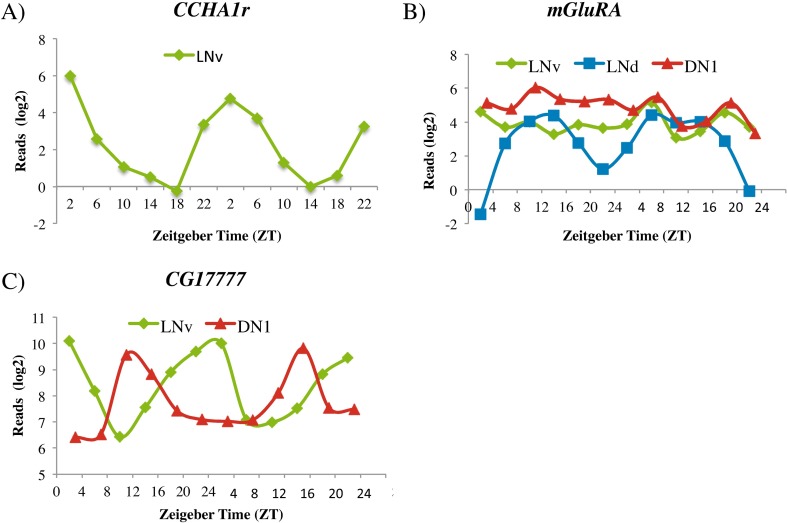
Three transcripts that show neuron-specific cycling patterns. In all figures, LNvs are shown in green, LNds are shown in blue and DN1s are shown in red. A) The transcript encoding the G-protein coupled receptor, *CCHA1r*, cycles in the LNvs with peak expression in the morning. *CCHA1r* transcripts are not expressed or expressed at very low levels (<5 average reads/million) in LNds and DN1s. B) The transcript encoding the metabotropic glutamate receptor (*mGluRA*) cycles robustly in the LNds with peak expression at mid-day. *mGluRA* is expressed at constant levels in both the LNv and DN1s. C) The transcripts encoding the predicted neuropeptide *CG17777* cycle with different phases in the LNvs and DN1s. *CG17777* is also expressed in the LNds but does not cycle.

Most cycling mRNAs are specific to a single group of circadian neurons (Fig 4B and S2 Fig). This is partially explained by differential gene expression: ~15% of neuron-specific cycling transcripts are not expressed in the other two neuronal groups. For example, *CCHa1r* mRNA encodes a GPCR and is one of the top LNv cyclers; it peaks in the morning (ZT2), disappears at night (Fig 5A) and is not expressed in either LNds or DN1s (definition: average expression >5 reads/million).

The remainder, ~85% of the clock neuron cycling transcripts, oscillate specifically in one group despite being expressed in one or both of the other circadian groups. For example, the metabotropic glutamate receptor (*mGluRA*) mRNA is one of the highest amplitude cyclers in LNds, ~60-fold [28]. Although it only cycles in LNds (Fig 5B), *mGluRA* mRNA is also expressed at comparable levels in LNvs and DN1s as previously reported [54]. This suggests that LNvs and DN1s also respond to glutamate but only LNds temporally modulate their response to this neurotransmitter [28].

There are also a few examples in which a transcript may cycle in two groups of circadian neurons but with a different phase. *CG17777* mRNA encodes a putative novel neuropeptide and is a HC cycler in LNvs and DN1s (Fig 5C). These transcripts peak in the early morning and are at their nadir during the mid-day in LNvs, but they are lowest in early morning and peak at mid-day in DN1s. It is also expressed in LNds, where it does not cycle.

**Fig 6:**
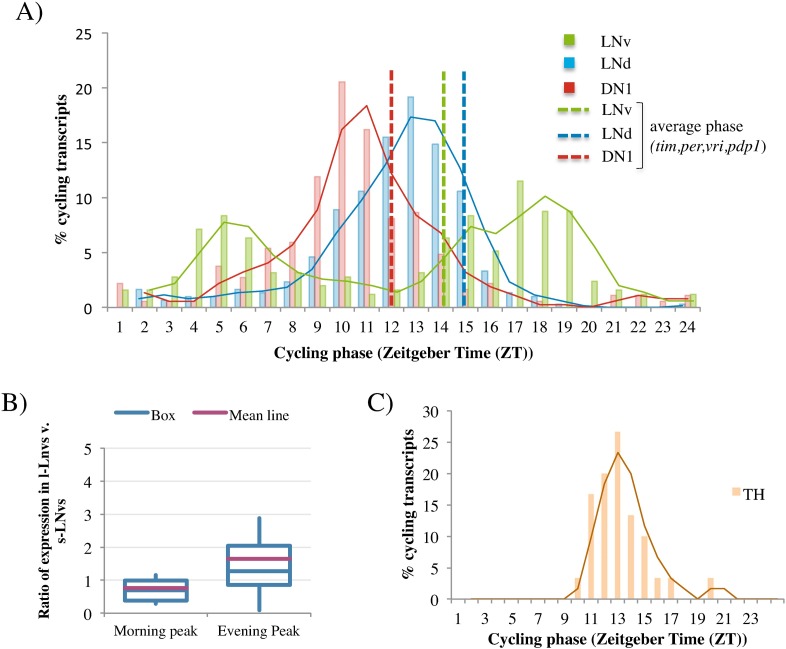
Transcripts cycling in LNvs show bimodal phase distribution. A) The phase of all the high confidence (HC) cycling transcripts identified in each neuronal group is represented in a histogram (LNvs (green), LNds (blue), DN1s(red)). The transcripts are binned according to their peak expression (phase) and the percentage of all cycling transcripts in that bin is plotted. The shape of the distribution is emphasized by a trendline in the same color as the histogram. The lines indicate the average phase of the core clock genes (*vri*, *pdp1*, *per*, *tim*) for each of the three neuronal groups. Phase shown was calculated by F24 analysis. B) For every cycling transcript in LNvs, the relative expression in l-LNvs versus s-LNvs was calculated (see Methods). Those transcripts cycling with a morning (ZT3-8) and evening phase (ZT14-ZT19) were binned and the distribution of expression in l-LNvs versus s-LNvs was visualized using a box plot. Transcripts cycling with a morning phase were more likely to be more highly expressed in s-LNvs (relative expression large v. small <1) and transcripts cycling with an evening phase are more likely to have higher expression in l-LNvs (relative expression large v. small >1). These two different distributions are significantly different (p-value: 3x10^-5^). C) The phase of all the HC cycling transcripts in dopaminergic (TH) cells. Data representation is the same as in (A).

A comparison of cycling phase between neuron groups was also done genome-wide despite the fact that most transcript cycling is restricted to a single group. To this end, the three HC cycling transcript phase distributions were plotted as histograms, i.e., % of all cyclers with a particular phase (Fig 6A). Cycling transcripts in LNds and DN1s have similar unimodal phase distributions centered around mid-day (Fig 6A, red and blue). It is difficult to determine whether these two distributions are truly different. This is because the time points used for the DN1 and LNd purifications were somewhat different, which could modestly affect phase determination (see Methods). However, the cycling LNv transcripts have a very different distribution; it is bimodal with one peak shortly after lights on and the other shortly after lights off (Fig 6A; green). This LNv bimodal phase distribution is maintained with the inclusion of LC as well as HC cyclers (900 transcripts in total; S3 Fig).

In contrast, the CLK/CYC direct target core clock transcripts (*tim*, *per*, *vri*, *pdp1*) have a similar unimodal phase of ~ZT12-ZT15 in all three groups. (Fig 6A; red, blue, and green dotted lines). This phase is also similar to what has been observed in heads for these same transcripts [55]. As ZT12-15 is very different from the overall cycling transcript phase distribution from LNvs, it indicates that this different profile is unlikely due to a technical or analytical artifact. This suggests in turn that the different phase distributions reflect at least two different mechanisms operating in the three clock neuron groups (see Discussion).

One simple hypothesis is that the bimodal phase distribution comes from the two functionally distinct subclasses of LNv neurons: the s-LNvs and the l-LNvs. As the s-LNvs have been shown to track lights-on [56], we considered that those transcripts peaking in the morning might come predominantly from s-LNvs (Fig 6B). To address this possibility, we used previous microarray data that separately profiled the s-LNvs and l-LNvs to calculate the expression ratio of each transcript in the two clock neuron groups [40]. The distribution (a value of <1 indicates more expression in s-LNvs; Fig 6B) indeed indicates that transcripts peaking in the morning show higher expression in the s-LNvs than transcripts peaking in the evening (t-test; p-value 3x10^-5^; see Discussion).

Given the low number of TH neuron HC cyclers (31), they may be false positives. Interestingly however, their phase distribution is not random but is centered around mid-day (Fig 6C). As this distribution anticipates the light-dark transition, it suggests a regulated mechanism of transcriptional regulation within TH neurons rather than a response to the light-dark cycle (see Discussion). In addition, gene ontology analysis indicates that TH cyclers are enriched in interesting functions including the mitochondrial inner membrane (HC cyclers as well as all cyclers (HC and LC)), cytochrome-c oxidase activity (all cyclers), and the chaperone tailless complex polypeptide 1 (TCP1; all cyclers).

## Discussion

We have profiled the transcripts from three circadian neuron groups as well as from TH neurons. The profiling was also done as a function of time so that transcript oscillations as well as relative transcript abundance could be assessed in the four groups.

In TH neurons, some clock gene mRNAs like *Clk* are difficult to detect, and all clock gene expression is at least ~5x lower than in the circadian neurons. This is also the case for *tim* mRNA, which is the only core clock gene (LC) cycler in TH neurons. The low expression levels and weak cycling of clock gene mRNAs suggest a substantial quantitative and probably qualitative difference in gene expression between the 75 pairs of clock neurons and TH neurons. The low core clock gene RNA levels in TH neurons could also reflect some clock neuron contamination or that a small percentage of the TH neurons contain a molecular clock. However, we have evidence that the default state of general brain neuron chromatin is permissive for a low level of non-cycling core clock gene transcription (S4 Fig), which is mechanistically distinct from clock gene expression in circadian cells. All of these data suggest that TH neurons do not express a functional circadian clock. Indeed, this notion is consistent with previous immunostaining experiments showing that CLK is detectable only in the ~150 circadian neurons in the adult brain [57].

The absence of an endogenous clock can also explain why TH neurons have many fewer cycling transcripts. Although they too could be due to contamination or false positives, these cyclers are enriched in specific functions. Their striking phase distribution (Fig 6C) also suggests that they are genuine cyclers and further suggests that this cycling is governed by a single prominent mechanism. These rhythms could be an indirect consequence of behavioral or physiological rhythms (homeostatic regulation) or be modulated by the 150 circadian neurons. This interpretation recalls results from mammals in which 10% of cycling liver transcripts are driven by systemic cues and continue to cycle even in the absence of a clock in the liver [58]. Interesting, the percentage of TH cyclers compared to those in the circadian neurons is not dissimilar, i.e., 5–10% (Fig 4B).

It is possible that this view extends to mammals, i.e., that not all mammalian cells and most importantly not all brain neurons contain a functional circadian clock. Mammalian transcripts important for dopamine synthesis such as tyrosine hydrolyase (TH) oscillate throughout the day in dopaminergic neurons [59, 60]. Although mammalian dopaminergic neurons in the ventral tagmental area (VTA) have been reported to express CLK [59], recent neuron-specific transcriptome studies report little or no core clock gene expression in dopaminergic neurons (personal communication, S. Nelson; Neuro-seq project). It is therefore possible that transcript cycling in mammalian dopaminergic neurons is also driven by signals from elsewhere in the brain, perhaps from the SCN, and that a functional clock may not exist in all mammalian cells.

There is substantial overlap between this RNA-seq profiling of clock neurons and previous microarray experiments [37, 40]; PDF neurons, s-LNvs and l-LNvs, as well as the overall clock neuron population were separately characterized in these studies. Moreover, several brain proteins, including neuropeptides and neurotransmitter systems, have been previously identified within different clock neurons by immunohistochemistry. These results also agree for the most part with our transcript profiling, suggesting that the neuron purification and RNA sequencing libraries properly describe the transcriptomes of the 3 clock neuron groups. The few discrepancies indicate clock neurons with specific mRNAs but no detectable protein. Although this could reflect contamination, a more positive interpretation is that some cell-type specific protein expression may rely on post-transcriptional regulation. The neuropeptide Dh31 is a good example: its transcript has a shorter 3’UTR in LNvs than in DN1s, which argues strongly against contamination with DN1 RNA (Fig 4C). As the neuropeptide is detectable in DN1s but not LNvs, the longer 3’UTR may be necessary for the binding of required RNA binding proteins, for example positive translation factors.

In contrast to LNvs, LNd and DN1 genome-wide profiling has not been previously reported. These groups include cells that promote evening activity and sleep, respectively [19, 28]. Consistent with these behavioral roles, LNds cells have enriched levels of acetylcholine enzyme mRNAs, whereas DN1s have enriched levels of the glutamate vesicular transporter (*Vglut;* Fig 2D) mRNA. (Glutamate acts as an inhibitory neurotransmitter in the circadian network; [28] These transcripts are functional: RNAi of the acetylcholine vesicular transporter mRNA in LNds cells increases sleep (S5 Fig), whereas RNAi of the glutamate vesicular transporter within DN1s reduces sleep [28]

The LNds and DN1s also contain neuropeptide transcripts not previously implicated in the circadian system. Although the functions of most of these neuropeptides are not understood, PK-2 (encoded by the propeptide *hugin)* is implicated in feeding control [61]. This suggests that the circadian system may use PK-2 to convey time of day information to neurons modulating feeding. PK-2 and several other identified peptides (Dms and CNMa), have mammalian homologs that may have a role in the mammalian circadian system. For example, the PK-2 homolog Neuromedin-U (NmU) is regulated by the circadian clock in the SCN [62]. Some of these neuropeptides as well as additional neurotransmitters and neuropeptide receptors (Fig 3A) may also contribute to cell-specific circadian functions in *Drosophila*.

These neuropeptides are representative of most differential and cycling gene expression; the three different circadian neuron groups are largely distinct. We expected the profiles to be more shared, but only the core clock genes and a handful of additional genes are regulated similarly in the three clock neuron groups. Even with relaxed criteria to include a greater number of cycling transcripts from each cell group, there was no change to the conclusion, nor was it changed by including LC as well as HC cyclers. However, the limited overlap in cycling transcripts could be influenced by cell heterogeneity, which exists within each group. The DN1s have the greatest number of enriched mRNAs and may be the most variable of the 3 groups (S2 File). They likely contain sleep-promoting cells expressing glutamate as well as arousal-promoting cells containing Dh31 [25, 28].

These cell-type specific cycling gene expression results recall similar comparisons between mammalian tissues: cycling gene expression is predominantly tissue-specific with only modest shared gene expression beyond the core clock genes [51, 63]. This interpretation also offers a simple explanation of why most of these cycling transcripts were absent from those previously reported in fly heads [47–50]: most neuron-specific cycling transcripts are obscured by the same non-cycling transcript from many other neurons in head mRNA.

Despite the different cycling mRNAs, those in LNds, DN1s and TH cells have similar phase distributions; they peak at about ZT11-13. This is similar to the well-described phase of CLK/CYC-controlled gene expression from heads [55]. In contrast, the LNv phase distribution is dramatically different: it has two peaks, one shortly after lights-on at ZT0 and the other shortly after lights-off at ZT12. The striking difference between the LNvs and the other circadian neurons is unlikely due to technical or analytical difficulties, as the CLK/CYC controlled core clock mRNAs in LNvs have a similar unimodal phase at around ZT14 like the other 2 circadian groups. In addition, we observe the same bimodal phase distribution when including LC as well as HC cyclers (S3 Fig).

It is tempting to assign the two peaks of cycling transcripts to the s-LNvs and the l-LNvs, respectively. Indeed, transcripts with the later phase show a statistically significant bias toward higher expression in the l-LNvs rather than the s-LNvs (Fig 6B). However, there are multiple exceptions, e.g., genes in the evening peak that were previously found to be more highly expressed in s-LNvs. Although intragroup heterogeneity complicates the interpretation, there are probably two major peaks of expression/day even within a single cell type, which is similar to data from mammalian liver and SCN [51, 64]. However, these are different transcripts, i.e., we do not reliably detect a group of transcripts with two peaks/day comparable to the light sensing pathway recently reported from the SCN [51]. This difference may reflect the major differences in light sensing and light input pathways between flies and mammals. Stronger conclusions will require more experiments.

The mechanisms that underlie these cell type-specific phase distributions are unknown. The similarity between LNds and DN1s suggests that they share common mechanisms if not molecules, which likely differ in PDF cells. We can imagine two possibilities to explain the similar LNd and DN1 patterns. One is that these two groups share a circadian firing pattern, which results in a common circadian pattern in calcium and calcium-dependent gene expression. However, recent results suggest that the calcium activity patterns of LNds and DN1 are quite different, with the LNds firing in late morning and the DN1s at late night and early morning [21, 56]. The other is that the two groups receive similar circadian input, for example from light or from PDF activation of the PDF receptor (PDFR). As PDF signaling is under circadian control [5, 65, 66], it should result in a similar circadian signal transduction pathway downstream of PDFR [67]. This could give rise to a common phase of cycling gene expression in LNds and DN1s despite substantial differences in responsive (accessible) genes. The connection of the LNvs with light input suggests that its very different phase distribution might reflect a gene expression response to the lights-on and lights-off stimuli characteristic of the entrainment protocol. All of these possibilities require experimental support and still do not address the mechanisms or molecules that underlie the phase distributions within PDF neurons.

## Methods

### Flies

In order to visualize neurons for sorting the following fly lines were used: *Pdf-GAL4*, UAS-*mCD8*::*GFP* for LNvs, Dv-*Pdf*-*GAL4*, UAS-*EGFP*, *PDF-RFP* for LNds, yw; *CLK4*.*1m*-*GAL4*, UAS-*EGFP* for DN1s and yw; UAS-*EGFP*; *TH-GAL4* for dopaminergic or TH cells. *Pdf-RFP* flies were a gift of J. Blau. *CLK*^*out*^ flies were a gift of P. Hardin [68]. *ChAT* RNAi flies were obtained from Bloomington Drosophila Stock Center (BL25856, [69]).

### RNA extraction and amplification for sequencing libraries

Flies were entrained for 4 days in 12:12 LD cycles. Fly brains were isolated every 4 hours for two independent sets of six circadian timepoints for each neuron group. Samples for LNvs, LNds and TH were collected at ZT2, ZT6, ZT10, ZT14, ZT18 and ZT22. Samples for DN1s were collected at ZT3, ZT7, ZT11, ZT15, ZT19 and ZT23. Brains were dissociated and the neurons of interest were isolated using three rounds of manual sorting using a fluorescent microscope. PolyA+ RNA was isolated from approximately 50–100 isolated neurons and subjected to one round of linear amplification prior to making libraries for deep sequencing [30]. Libraries were sequenced on a Hi-Seq 2000 (Illumina) using 50bp single end reads.

RNA for whole brain RNA-seq was extracted from brains collected at ZT2 and ZT14 using standard Trizol methods (Invitrogen). Libraries were made following the standard protocol of the TruSeq RNA Sample Prep Kit (v2; Illumina).

### Analysis of sequencing data

The resulting sequencing files were mapped to the *Drosophila* genome (dm3) using Tophat [70, 71]. On average ~50% of the reads mapped to the genome. Lower mapping frequencies were due to a number of factors including the presence of rRNA and contamination of the libraries with non-*Drosophila* nucleic acid. The total number of reads in each library is summarized in S1 File. The libraries generated from small numbers of purified neurons show 3’-bias (Fig 1). Although sometimes recommended, we did not remove identical sequencing reads (often called removing PCR duplicates) from our sequencing libraries since oligo-dT amplification lead to an abundance similar 3’-reads in the libraries that would be removed. After mapping, gene expression was quantified using End Sequencing Analysis Toolkit (http://garberlab.umassmed.edu/software/esat/; [72]. ESAT quantitates gene expression by examining reads in a sliding 300bp window at 3’-end all isoforms of a gene and prevents any bias introduced by differences in gene length using more standard methods such as Cufflinks [73]. To ensure that gene expression is quantified similarly and is comparable, all 48 libraries were analyzed simultaneously using ESAT. Gene expression values were normalized and are expressed as reads per one million reads. As noted in earlier studies, the low amount of starting material isolated from purified neurons leads to lower sample reproducibility than observed with typical RNA-seq experiments [74]. The mean values for Pearrson coefficients for pair-wise sample comparisons for LNvs, LNds, DN1s and TH neurons were 0.9, 0.83, 0.87 and 0.81, respectively. There was typically more variation from libraries from dopaminergic cells perhaps due to the larger amount of heterogeneity in that population (~120 TH cells in the brain; [75]).

To visualize transcript levels in the sequencing libraries, bigwig visualization files were made from bam files and were visualized using the integrated genome browser (IGV; Whitehead Institute). In Fig 1, the images represent the sum of all 12 samples made from each cell type. To illustrate cycling, replicate timepoints were combined to more concisely show cycling transcript levels (Fig 4C). Heatmaps were produced from normalized expression data using heatmap.2 in gplots package for R. Sequencing data is available at Gene Expression Omnibus (Accession number GSE77451).

### Identification of differentially expressed genes

To identify differentially expressed genes, the average transcript levels was calculated for each set of 12 samples in each neuronal group (two 6 timepoint circadian experiments). Transcripts expressed at low levels were removed by requiring an average of at least 10 reads/million in each of the two independent six timepoint experiments. The relative difference in transcript level in each of the four neuronal groups was calculated by taking the ratio of the averages. Transcripts that showed a 2-fold change in levels were analyzed further for statistical significance. An Anova analysis was performed with a p-value cutoff of 0.05. A Tukey HSD post-hoc analysis was used to identify statistically significant groups and a Benjamini Hochberg correction (p-value <0.05) was used to account for the complications of multiple comparisons. Transcripts were considered to be enriched in circadian neurons if they showed a 5-fold enrichment when compared to TH neurons in at least 2 of the circadian neuron groups and met the statistical cutoffs. Transcripts were considered to be specifically enriched in one group of circadian neurons if they met all statistical cutoffs and were > 5-fold higher relative to one circadian neuron group and >2-fold higher relative to the other.

Gene Ontology analysis was performed using DAVID bioinformatics resources [76, 77]. In all cases analyses were performed using a list of neuron-specific genes as a background comparison. A p-value of less than 0.05 was required in order for a gene ontology classification to be considered enriched.

### Identification of potential neuropeptides using NeuroPID

To identify potential neuropeptides that play a role in the circadian system, we identified those genes that were more highly expressed in each group of circadian neurons relative to the whole brain (data for brains from [53]. The peptide sequence of all genes with higher expression in circadian neurons was obtained using FlyMine [78] and submitted to NeuroPID [46] http://neuropid.cs.huji.ac.il. NeuroPID was used to identify putative neuropeptide precursors that contained candidate signal peptides and were identified as high confidence predictions. NeuroPred (http://stagbeetle.animal.uiuc.edu/cgi-bin/neuropred.py) was then used to explore the cleavage sites of these neuropeptide precursors. A subset of those novel neuropeptide precursors that were identified were included in Fig 3A.

### Identification of cycling transcripts

To identify cycling transcripts, normalized transcript levels for two independent experiments (6 timepoints each) generated by ESAT were used as input. Transcript expression values were normalized relative to the maximum signal in each set of 6 timepoints as previously described [79]. Cycling transcripts were identified using both fourier transformation [79] and JTK_cycle [80]. To be considered cycling using fourier transformation the following cutoffs were used: F24 score greater than 0.5, >2 fold amplitude of transcript cycling, and the average transcript reads greater than 5. JTK_cycle identified transcripts as cycling that had a >2 fold amplitude of transcript cycling, average transcript reads greater than 5, and a p-value cutoff of less than 0.05. The overlap of these two approaches ranged from 30–80% (Sup Fig 1). Those cycling transcripts identified by both methods were considered high-confidence cyclers (HC cyclers) and those identified by only one method were considered low-confidence cyclers (LC cyclers).

To examine whether LNv cycling transcript expression came primarily from the s-LNvs or l-LNvs, we utilized data from previously microarray studies [40]. The ratio of expression in l-LNvs versus s-LNvs was calculated and used as a metric for expression in the two neuronal subtypes.

## Supporting information

S1 File Excel file describing sequencing files and mapped reads. (XLSB)

S2 File Excel file describing transcripts enriched in LNds, LNvs, and DN1s. (XLSX)

S3 File Excel file describing transcripts that cycle in LNds, LNvs, DN1s and TH neurons. (XLSX)

S1 Fig Intersection of cycling transcripts identified by Fourier transformation or JTK cycle. Cycling transcripts identified by Fourier transformation (orange) or JTK cycle (light blue) are shown for each neuronal group. High confidence (HC) cycling transcripts found by both methods are shown in gray. Transcripts identified by either Fourier transformation or JTK cycle but not both methods were considered low confidence (LC) cyclers.
(PDF)

S2 Fig Many transcripts cycle in a single group of circadian neurons. The expression values for transcripts found to be cycling in one subset of the circadian neurons are represented using a heatmap. Cycling is clear in one set of circadian neurons and absent in the others. Low values are shown in blue (less than 30% of maximum signal), mid values are shown in black (between 30 and 60% of maximum signal) and high values are shown in yellow (greater than 70% of maximum signal).
(PDF)

S3 Fig Bimodal phase distribution of transcripts cycling in LNvs is maintained when low-confidence (LC) as well as high-confidence (HC) cyclers are included. A total of ~900 HC and LC cycling transcripts are binned according to their peak expression (phase) and the percentage of all cycling transcripts in that bin is plotted. The shape of the distribution is emphasized by a trendline in the same color as the histogram. Phase shown was calculated by F24 analysis.
(PDF)

S4 Fig Low levels of *tim* and *per* transcripts are detected via brain RNA seq from *Clk^out^* flies. Brain RNA-seq libraries were made from both wild-type (w118) and *Clk* deletion (*Clk*^*out*^) flies. The resulting data is shown using the integrated genome viewer (IGV). *Clk* transcripts are high at ZT2 and low at ZT14 in w118 flies, and not detectable in Clk^out^ flies (top). *Tim* and *Per* transcripts are low at ZT2 and high at ZT14 in w118 brains (as expected). Interestingly, both *tim* and *per* transcripts are still detectable at low levels (similar to that observed at ZT2 in w118 flies) in *Clk*^*out*^. This suggests that low levels of *Tim* and *Per* transcripts are made even in the absence of CLK.
(PDF)

S5 Fig *ChAT* expression in the LNds is important for sleep suppression. Transcripts encoding choline acetyltransferase (*ChAT*) are enriched in LNds. Knockdown of *ChAT* transcripts specifically in the LNds (*Dv-PDF-GAL4*, *pdfgal80; pdfgal80* driver) using RNAi results in an increase in total sleep (black). This increase is statistically significant (p-value <0.05 by one-way Anova) compared to both the driver only control (white) and the RNAi only control (gray). n = 16 for all genotypes.
(PDF)
