# Supplementary material for: RNA-seq analysis of Drosophila clock and non-clock neurons reveals neuron-specific cycling and novel candidate neuropeptides
Source: PLoS Genet. 2017 Feb 9;13(2):e1006613. doi: 10.1371/journal.pgen.1006613 (PMC5325595; doi:10.1371/journal.pgen.1006613)
Supplement: S1 Fig — Cycling transcripts identified by Fourier transformation (orange) or JTK cycle (light blue) are shown for each neuronal group. High confidence (HC) cycling transcripts found by both methods are shown in gray. Transcripts identified by either Fourier transformation or JTK cycle but not both methods were considered low confidence (LC) cyclers. (PDF) [file pgen.1006613.s004.pdf]

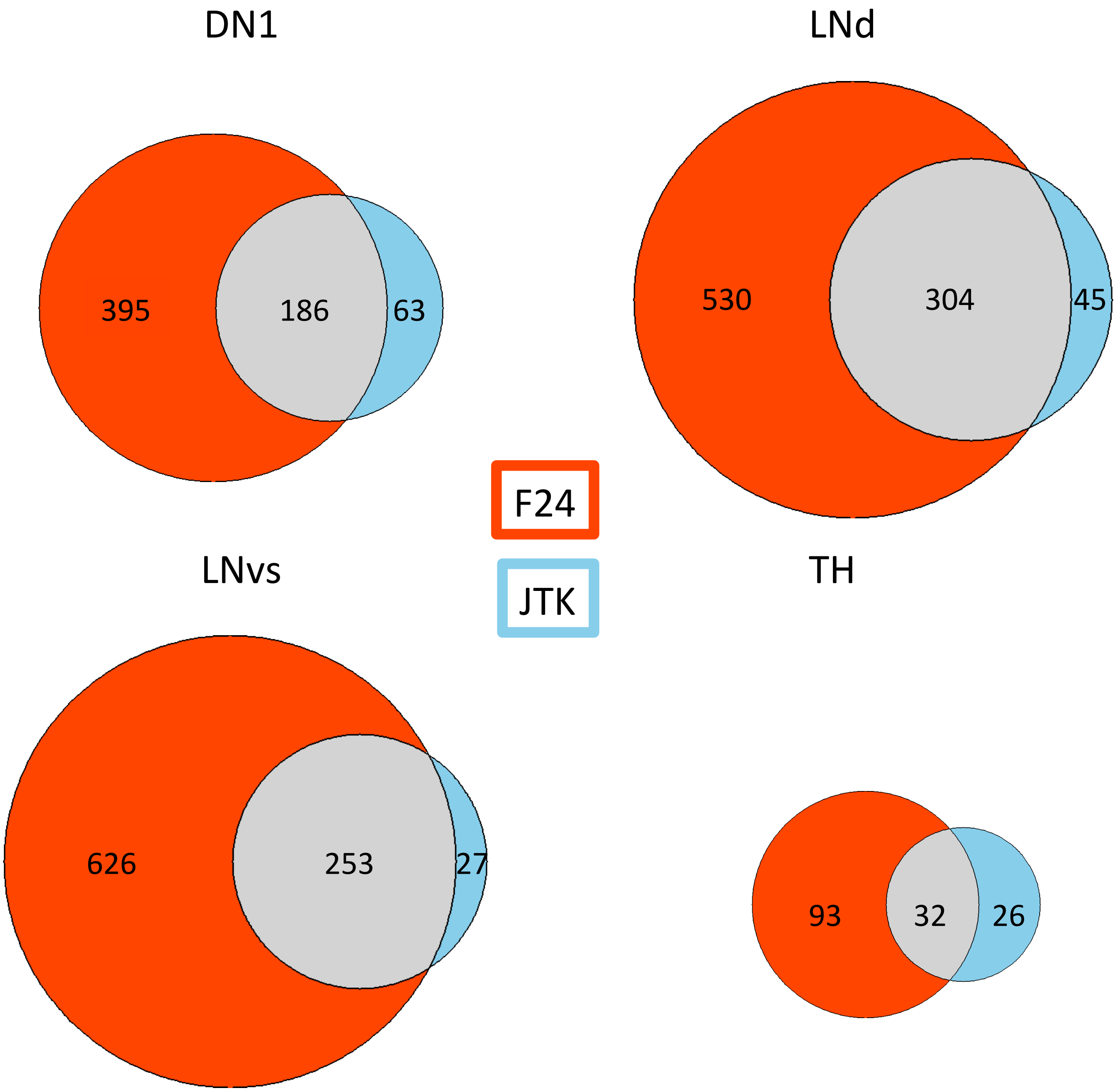

Supporting Figure 1. Intersection of cycling transcripts identified by Fourier transformation or JTK cycle. Cycling transcripts identified by Fourier transformation (orange) or JTK cycle (light blue) are shown for each neuronal group. High confidence cycling transcripts found by both methods are shown in gray. Transcripts identified by either Fourier transformation or JTK cycle but not both methods are considered low confidence cyclers.
