# Supplementary material for: RNA-seq analysis of Drosophila clock and non-clock neurons reveals neuron-specific cycling and novel candidate neuropeptides
Source: PLoS Genet. 2017 Feb 9;13(2):e1006613. doi: 10.1371/journal.pgen.1006613 (PMC5325595; doi:10.1371/journal.pgen.1006613)
Supplement: S2 Fig — The expression values for transcripts found to be cycling in one subset of the circadian neurons are represented using a heatmap. Cycling is clear in one set of circadian neurons and absent in the others. Low values are shown in blue (less than 30% of maximum signal), mid values are shown in black (between 30 and 60% of maximum signal) and high values are shown in yellow (greater than 70% of maximum signal). (PDF) [file pgen.1006613.s005.pdf]

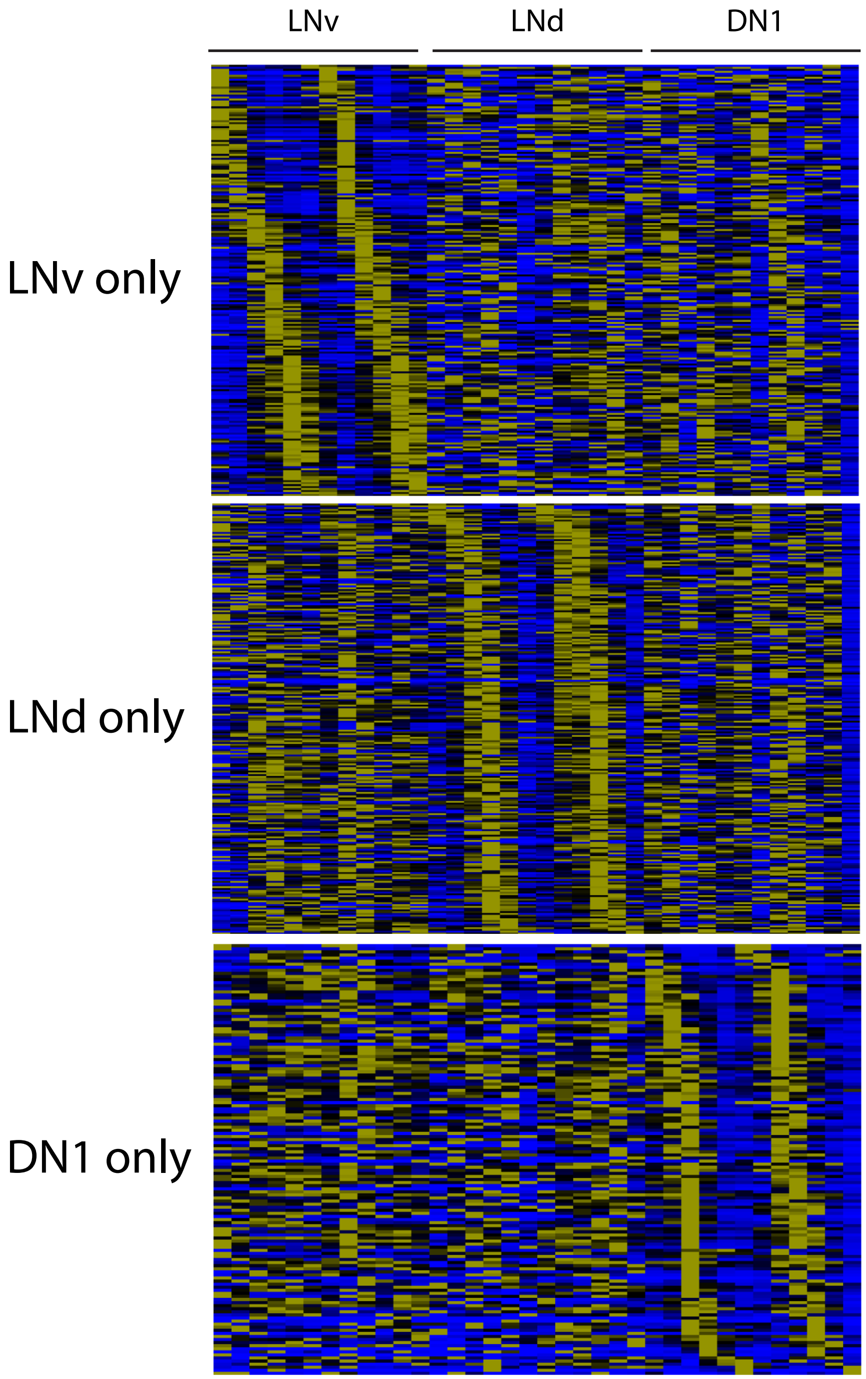

Supporting Figure 2. Heatmap representing expression patterns of transcripts found to be cycling in one group of circadian neurons. The expression values for transcripts found to be cycling in one subset of the circadian neurons are represented using a heatmap. Cycling is clear in one set of circadian neurons and absent in the others. Low values are shown in blue (less than 30% of maximum signal), mid values are shown in black (between 30 and 60% of maximum signal) and high values are shown in yellow (greater than 70% of maximum signal).
