# Supplementary material for: RNA-seq analysis of Drosophila clock and non-clock neurons reveals neuron-specific cycling and novel candidate neuropeptides
Source: PLoS Genet. 2017 Feb 9;13(2):e1006613. doi: 10.1371/journal.pgen.1006613 (PMC5325595; doi:10.1371/journal.pgen.1006613)
Supplement: S3 Fig — A total of ~900 HC and LC cycling transcripts are binned according to their peak expression (phase) and the percentage of all cycling transcripts in that bin is plotted. The shape of the distribution is emphasized by a trendline in the same color as the histogram. Phase shown was calculated by F24 analysis. (PDF) [file pgen.1006613.s006.pdf]

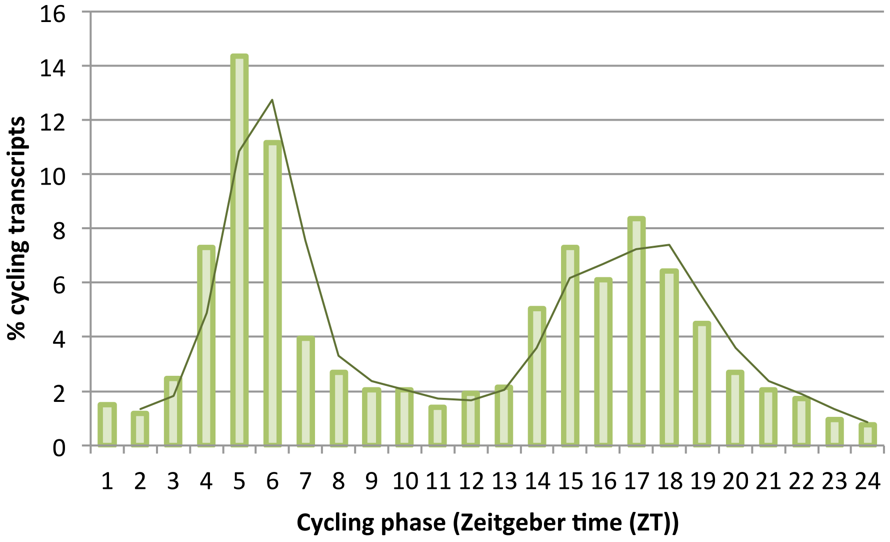

Supporting Figure 3. Bimodal phase distribution of transcripts cycling in LNvs is maintained when low-confidence (LC) as well as high-confidence (HC) cyclers are included. A total of ~900 HC and LC cycling transcripts are binned according to their peak expression (phase) and the percentage of all cycling transcripts in that bin is plotted. The shape of the distribution is emphasized by a trendline in the same color as the histogram. Phase shown was calculated by F24 analysis.
