# Supplementary material for: RNA-seq analysis of Drosophila clock and non-clock neurons reveals neuron-specific cycling and novel candidate neuropeptides
Source: PLoS Genet. 2017 Feb 9;13(2):e1006613. doi: 10.1371/journal.pgen.1006613 (PMC5325595; doi:10.1371/journal.pgen.1006613)
Supplement: S4 Fig — Brain RNA-seq libraries were made from both wild-type (w118) and Clk deletion (Clkout) flies. The resulting data is shown using the integrated genome viewer (IGV). Clk transcripts are high at ZT2 and low at ZT14 in w118 flies, and not detectable in Clkout flies (top). Tim and Per transcripts are low at ZT2 and high at ZT14 in w118 brains (as expected). Interestingly, both tim and per transcripts are still detectable at low levels (similar to that observed at ZT2 in w118 flies) in Clkout. This suggests that low levels of Tim and Per transcripts are made even in the absence of CLK. (PDF) [file pgen.1006613.s007.pdf]

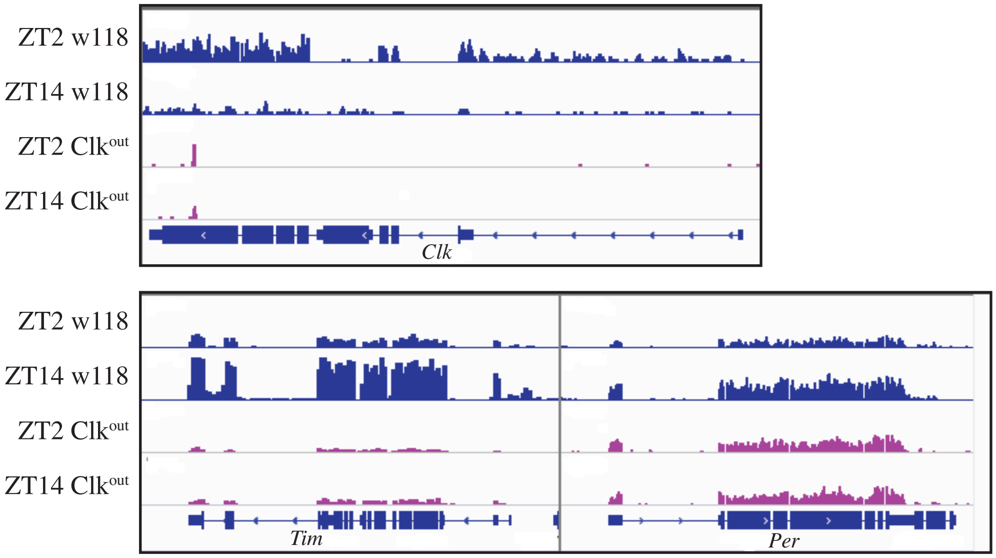

Supporting Figure 4. Low levels of *tim* and *per* transcripts are detected via brain RNA seq from *Clk^out* flies. Brain RNA-seq libraries were made from both wild-type (w118) and *Clk* deletion (*Clk^out*) flies. The resulting data is shown using the Integrated genome viewer (IGV). *Clk* transcripts are high at ZT2 and low at ZT14 in w118 flies, and not detectable in Clk^out flies (top). *Tim* and *Per* transcripts are low at ZT2 and high at ZT14 in w118 brains (as expected). Interestingly, both *tim* and *per* transcripts are still detectable at low levels (similar to that observed at ZT2 in w118 flies) in *Clk^out*.
