# Supplementary material for: RNA-seq analysis of Drosophila clock and non-clock neurons reveals neuron-specific cycling and novel candidate neuropeptides
Source: PLoS Genet. 2017 Feb 9;13(2):e1006613. doi: 10.1371/journal.pgen.1006613 (PMC5325595; doi:10.1371/journal.pgen.1006613)
Supplement: S5 Fig — Transcripts encoding choline acetyltransferase (ChAT) are enriched in LNds. Knockdown of ChAT transcripts specifically in the LNds (Dv-PDF-GAL4, pdfgal80; pdfgal80 driver) using RNAi results in an increase in total sleep (black). This increase is statistically significant (p-value <0.05 by one-way Anova) compared to both the driver only control (white) and the RNAi only control (gray). n = 16 for all genotypes. (PDF) [file pgen.1006613.s008.pdf]

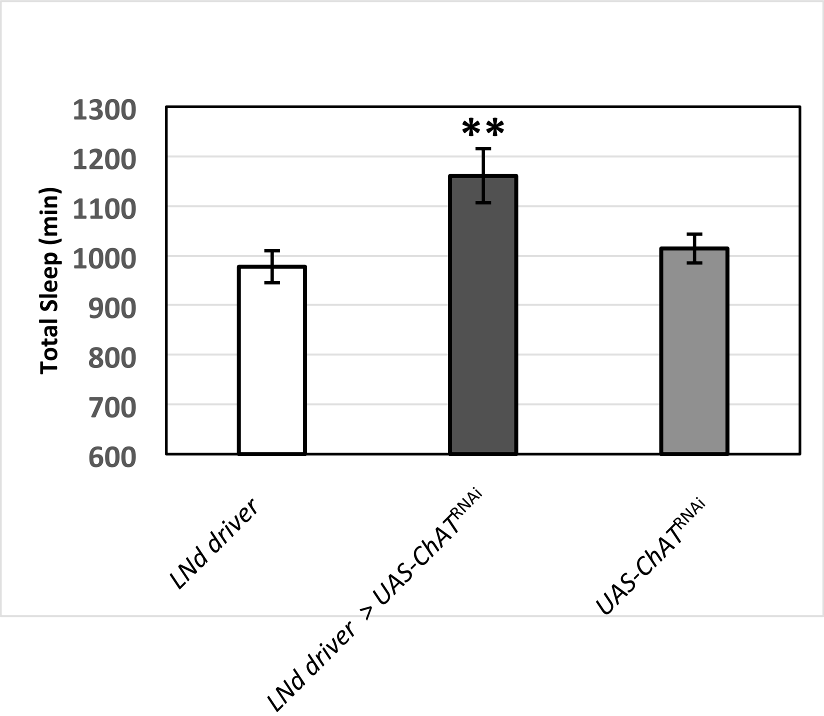

Supporting Figure 5. ***ChAT*** **expression in the LNds is important for sleep suppression.** Transcripts encoding choline acetyltransferase (*ChAT*) are enriched in LNds. Knockdown of *ChAT* transcripts specifically in the LNds (*Dv-PDF-GAL4, pdfgal80; pdfgal80* driver) using RNAi results in an increase in total sleep (black). This increase is statistically significant (p-value <0.05 by one-way Anova) compared to both the driver only control (white) and the RNAi only control (gray). n=16 for all genotypes.
